# CRISPR-Cas9-mediated labelling of the C-terminus of human laminin β1 leads to secretion inhibition

**DOI:** 10.1186/s13104-020-04956-z

**Published:** 2020-02-21

**Authors:** L. Shaw, R. L. Williams, K. J. Hamill

**Affiliations:** Institute of Ageing and Chronic Disease, University of Liverpool, 6 West Derby Street, Liverpool, L78TX UK

**Keywords:** Laminin, Basement membrane, Dendra2, Genome editing, CRISPR-Cas9

## Abstract

**Objectives:**

The laminins (LM) are a family of basement membranes glycoproteins with essential roles in supporting epithelia, endothelia, nerves and muscle adhesion, and in regulating a range of processes including cell migration, stem cell maintenance and differentiation. However, surprisingly little is known about the mechanisms of turnover and remodelling of LM networks due to lack of appropriate tools to study these processes at the necessary resolution. Recently, the nematode *C. elegans* ortholog of human the LMβ1 chain was labelled at the C-terminus with the photoconvertible fluorophore Dendra2. Here we used genome editing to establish a similar system in a mammalian cell line as proof of concept for future mammalian models.

**Results:**

CRISPR-Cas9 was used to introduce the Dendra2 sequence at the C-terminus of LMβ1 in the human lung adenocarcinoma cell line A549. Despite expression of the tagged protein within cells, no detectable LMβ1-Dendra2 protein was deposited to the extracellular matrices or conditioned media of edited cells. Moreover, the edited cells displayed reduced proliferation rates. Together, these data suggest that, in humans, addition of C-terminal Dendra2 tag to LMβ1 inhibits LM secretion, and is not a viable approach for use in animal models.

## Introduction

**Fig. 1:**
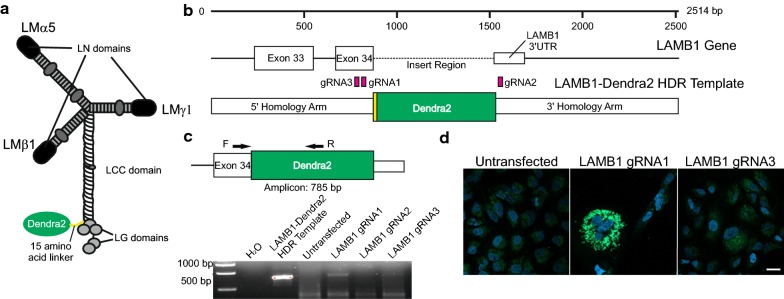
Design and transfection of LAMB1-Dendra2 HDR template into A549 cells. **a** Diagram of desired insertion of Dendra2. **b** Linearised sequence map of the LAMB1-Dendra2 HDR template donor, with gRNA protospacer adjacent motifs sites highlighted in pink and linker sequence in yellow. **c** Gel image from PCR performed on DNA from transfected cells and using primers designed to amplify only when the Dendra2 sequences was inserted in the appropriate genetic location. **d** Transfected cells fixed with a DAPI counterstain were imaged by confocal analysis. Scale bars represent 20 μm

Laminin (LM) are core components of all basement membranes (BMs) [1–3] and are essential for tissue function by providing a substrates for cell attachment and migration, a barrier to tumour invasion, and in regulating signalling pathways [4–6]. Each LM is an αβγ heterotrimer comprising one of five α chains, one of three β chains and one of three γ chains, each derived from a distinct gene [7]. LM heterotrimers assemble intracellularly via an α-helical laminin coiled coil (LCC) domain in each chain. In the α chains, the LCC is followed by five globular domains which contain the major cell-surface receptor binding sites while at the amino terminus of a subset of LMs are LN domains involved in LM network assembly (Fig. 1a) [8–12]. Despite extensive investigation, surprisingly little is known regarding the mechanisms and dynamics of LM deposition and remodelling, in part owing to lack of appropriate tools for viewing these nanoscale process in live conditions.

Recently, a series of elegant studies in *C. elegans* described genetically tagging the C-terminus of the worm LMβ chain ortholog with fluorescent proteins, allowing in vivo observation of BM turnover and remodelling [13, 14]. In human cells, adenoviral-mediated expression of the two smallest LMs, LMβ3 and LMγ2, with C-terminal fluorescent tags has also been performed [15, 16]. However, these constructs are at the adenoviral packaging limit and allow only transient expression. Advances in CRISPR-Cas9 genome editing present an opportunity to directly tag endogenous human LM genes for stable expression [17].

Here we aimed to establish an in vitro model to study human LM dynamics. We selected LMβ1 tagged with the photoconvertible protein Dendra2. Dendra2 emits green fluorescence under blue light; however, exposure to short wavelength light photo converts the protein from green to red [18]. Dendra2-tagged proteins can therefore be used for tracking protein dynamics, remodelling and turnover using super resolution microscopy.

## Main text

### Methods

#### Cell culture

A549 lung adenocarcinoma cells (ATCC^®^ CCL-185™) were maintained at 37 °C, 5% CO_2_ in high glucose (4.5 g/L) Dulbecco’s Modified Eagle Media with 2 mM l-glutamine (Sigma-Aldrich, St. Louis, Missouri, USA), supplemented with 10% foetal bovine serum (LabTech International Ltd, Heathfield, UK).

#### CRISPR-Cas9 genome-editing

A549 cells were transfected using 400 ng of one of three gRNA’s (gRNA1 ATAGCACATGCTTGTAACAG, gRNA2 AAAAATGGCTGAGGTGAACA, gRNA TTATATCCTTTAGGAGTGAA), 2 μg of Cas9 2×Nuclear Localisation Signal (GeneMill, Liverpool, UK), in a final volume of 7 μL of Neon™ R Buffer (ThermoFisher, Waltham, Massachusetts, USA). Cas9-gRNA solutions were incubated at room temperature for 20 min, 1.2 × 10^5^ A549 cells and 600 ng LMβ1-Dendra2 HDR donor template were added, and the solution electroporated using the Neon™ 10 μL Transfection Kit (ThermoFisher) with 4 × 20 ms 1200 V pulses then seeded onto pre-warmed 24-well plate.

#### Cloning and PCR screening

Populations were screened using PCR to detect the LAMB1-Dendra2 insert (forward primer TGGGTCTTTTCACACAGGCT, reverse CAGGGCCATGTTGATGTTGC, amplicon 785 bp). Single cell clones were generated by seeding 0.4 cells/well and expanding, then screened using PCR primers in LAMB1 exon 34 and 3′ untranslated region (3′UTR) (Forward GGAGAAGTCCGTTCACTCCT, reverse AAGGGATTCATCAACAATCAGTGA: 274 bp amplicon in non-edited cells, 967 bp with Dendra2 insert). 25 μL PCRs were run using 1 ng of genomic DNA, 1 μM primers, 12.5 μL REDTaq^®^ ReadyMix™ PCR Reaction Mix (Sigma-Aldrich), with the protocol; 95 °C for 5 min, 35 cycles of 95 °C for 30 s, 60 °C for 45 s and 72 °C for 1 min, ending with 7 min at 72 °C using a Veriti Dx Thermal Cycler™ (ThermoFisher). Products were separated by electrophoresis on a 2% agarose/TAE gel, and PCR bands purified using Monarch^®^ DNA Gel Extraction Kit (New England Biolabs, Ipswich, Massachusetts, USA) then sequenced by DNASeq (University of Dundee, Dundee, Scotland).

#### Immunoblotting

Cells were seeded in 100 mm tissue culture dishes for 24, 48, 72 or 96 h for a final population of 2 × 10^6^. Cells were scraped into 90 μL urea-SDS lysis buffer: 10 mM Tris–HCl pH 6.8, 6.7 M Urea, 1% w/v SDS, 10% w/v glycerol, 7.4 μM bromophenol blue, 50 μM phenylmethylsulfonyl fluoride, 50 μM *N*-methylmaleimide, sonicated and 10% v/v β-mercaptoethanol added (all Sigma-Aldrich). Conditioned media was collected and concentrated using a 40% w/v ammonium persulfate cut [19].

Samples were separated by SDS-PAGE on a 7.5% acrylamide/bis-acrylamide gel, transferred to 0.2 μm nitrocellulose membrane (Biorad, Hercules, California, USA) (100 V, 2.5 h), blocked for 1 h in 5% w/v Marvel^®^ Milk (Premier Foods, Hertfordshire, UK), then probed overnight at 4 °C with rabbit polyclonal antibodies against LMβ1 (1 μg/mL, ThermoFisher, PA5-27271). Membranes were washed 3 × 5 min in PBS with 0.1% Tween 20 (Sigma-Aldrich), and probed for 1 h at room temperature in the dark with IRDye^®^ 680IW conjugated goat anti-rabbit IgG secondary antibodies (0.05 μg/mL, LiCor Biosciences, Lincoln, Nebraska, USA). Membranes were washed for 3 × 5 min with PBS-Tween20 0.1%, rinsed with PBS then imaged using an Odyssey^®^ CLX 9120 infrared imaging system (LiCor Biosciences).

#### Immunocytochemistry and confocal microscopy

2 × 10^4^ cells were seeded on glass coverslips, then fixed using either 100% ice-cold methanol (University of Liverpool, UK) for 10 min and air-dried for staining with LMα5 antibodies (4C7, a gift from Prof Albrechtsen and Prof Wewer, University of Copenhagen, Denmark [20, 21]), or fixed in 3.7% v/v paraformaldehyde in PBS for 10 min and permeabilised in 0.1% v/v Triton X-100 (all Sigma-Aldrich) in PBS for 5 min. For ECM analyses, cells were removed by 2% v/v ammonium hydroxide treatment for 10 min prior to fixation [19]. Primary antibodies were diluted in 5% normal goat serum (Sigma-Aldrich) PBS and incubated at 37 °C for 2 h. Coverslips were washed 3 × 5 min with PBS then probed 1 h at 37 °C with Alexa Fluor^®^ 647 conjugated secondary antibodies (ThermoFisher) diluted in PBS at 2.4 μg/mL. Coverslips were washed in PBS with 0.05% Tween20 then mounted using VECTASHIELD^®^ Mounting Medium with DAPI (Vectorlabs, Murarrie, Australia) and fixed with nail varnish (Coco Chanel, Paris, France). Images were obtained using Zeiss LSM800 confocal microscope (Zeiss, Oberkochen, Germany), and processed using Zen Blue (Zeiss) and ImageJ (NIH, Bethesda, Maryland, USA).

#### Cell cycle analysis

Cells were serum starved for 24 h then seeded at a density of 2.5 × 10^5^ per well of a 6-well dish. 24 h later, cells were dissociated, pelleted, washed, fixed in 70% ethanol for 5 min, then resuspended in 150 µL of RNase A (0.5 mg/mL) (Sigma-Aldrich) and 150 µL of propidium iodide (ThermoFisher) (100 µg/mL) was added and incubated at 37 °C in the dark for 30 min. Cells were analysed using BD Accuri C6 flow cytometer (BD Biosciences, Franklin Lakes, New Jersey, USA). Multiple *T* test using the Holm-Sidak method was carried out using GraphPad Prism 6 (GraphPad Software, La Jolla, California USA).

### Results

#### Establishment of LMβ1::Dendra2 line

**Fig. 2:**
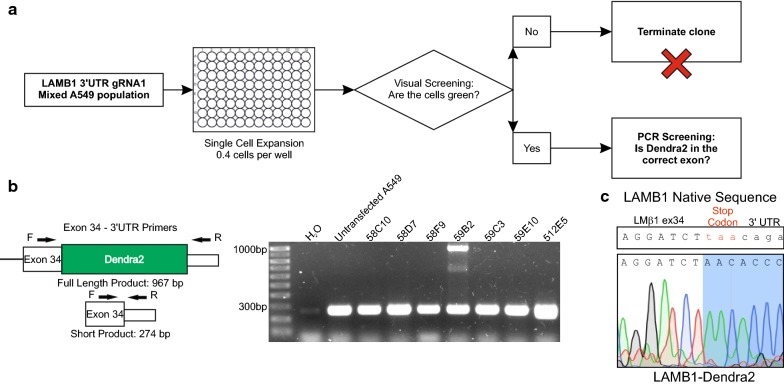
Establishment of the LMβ1::Dendra2 clonal line. **a** Diagram highlighting the screening workflow of > 500 single cell clones. Cells were expanded from single cells then screened based on green protein expression. Green clones were expanded then further screened using PCR. **b** Representative PCR gel from secondary screen on green clones using primers designed to give two potential products, a short (274 bp) and a full (967 bp) product in successfully edited cells only. **c** Positive clone 59B2 was sequenced to confirm for the presence of Dendra2. Start of the Dendra2 insert shown in SnapGene (LMβ1::Dendra2), with the sequence for exon34 and the 3′UTR of LMβ1 shown for reference

A549 cells were transfected with a LAMB1-Dendra2 HDR donor template, containing a 15 amino acid linker sequence GSGSNTPGINLIKED between the C-terminal of LMβ1 and Dendra2, equivalent to that used in *C. elegans* [14] (Fig. 1a). Three gRNAs were tested, each specific to different protospacer adjacent motif sites within exon 34 or the 3′UTR of LMβ1 (Fig. 1b), each of which had greater than three mismatches within other genes (Additional file 1). PCR using primers from LAMB1 (LMβ1 gene) exon 34 and 3′UTR from transfected cells showed a band matching the LAMB1-Dendra2 HDR template positive control for gRNA1 and a weaker band with gRNA3 (Fig. 1c). Cells were visually screened by imaging, which confirmed that gRNA1 had a higher proportion green cells compared to gRNA3 (Fig. 1d). gRNA1 was selected for single cell cloning (Fig. 2a): > 500 clonal populations were expanded and screened in a two-step procedure. First, by microscopy (Fig. 2a), then by PCR using primers designed to generate two potential products; 967 bp when Dendra2 was located between exon 34 and the 3′UTR, and 274 bp from non-modified LAMB1 (Fig. 2b). Despite screening hundreds of clones, this yielded only a single heterozygous clone, 59B2, containing the LAMB1-Dendra2 construct (Fig. 2b). DNA sequencing confirmed the higher band to be LAMB1-Dendra2 (Fig. 2c).

#### LMβ1::Dendra2 is expressed but not secreted from edited cells

**Fig. 3:**
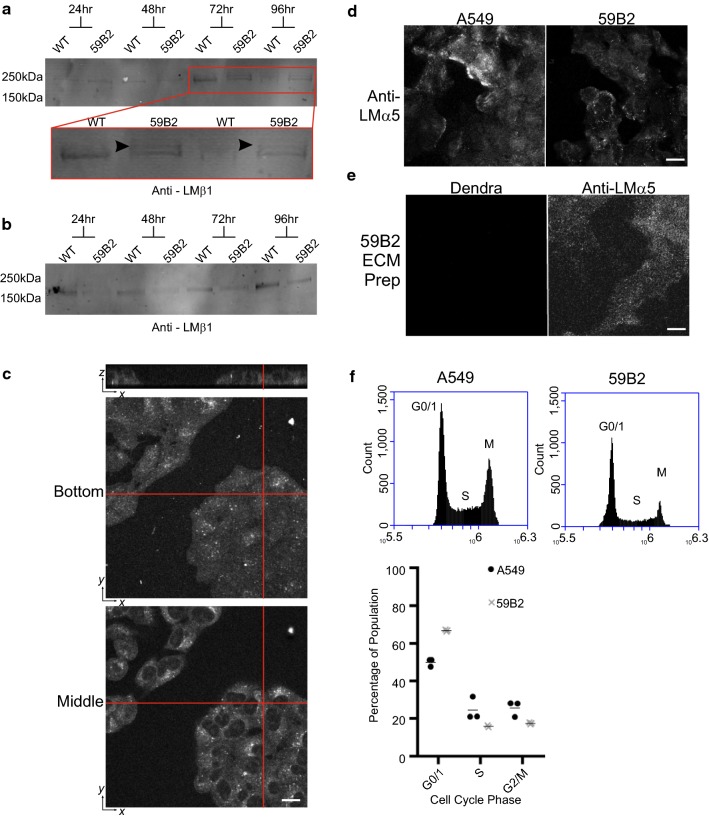
LMβ1::Dendra2 is expressed inside cells, but no secretion of LMβ1::Dendra2 was observed. **a** Total cell lysates were processed by western immunoblotting with anti-LMβ1 antibodies. Red box represents an enlarged section of the blot. Arrowed indicates additional upper LMβ1 band consistent with LMβ1::Dendra2. **b** Conditioned media and extracellular matrix lysates prepared for the indicated times then processed for immunoblotting with anti-LMβ1 antibodies. **c**, **d** Control A549 or 59B2 cells were seeded on coverslips for 48 h then either fixed and imaged with a z-stack (panel **c**) or processed for indirect immunofluorescence microscopy with antibodies against LMα5 (panel **d**). In **e** LMβ1::Dendra2 cells were cultured for 48 h on glass coverslips then removed with ammonium hydroxide to reveal only the extracellular matrix, then processed with antibodies against LMα5. **f** Cell cycle analysis of 59B2 cells (x’s) against wild type A549 cells (circles) analysed 24 h after serum shock. Proportion of cell population in each phase of the cell cycle was then plotted. Data was plotted in GraphPad. All scale bars represent 20 μm

To confirm expression of the Dendra2 tagged LMβ1 protein, cell and conditioned media extracts were collected from wild-type A549 cells and 59B2 LMβ1::Dendra2 cells: (Fig. 3a, b). Consistent with heterozygous expression of LMβ1::Dendra2, a second band approximately 20 kDa above the native LMβ1 band was obtained in cell extracts from the edited clone but not the controls (Fig. 3a). However, in conditioned media extracts, there was no evidence of the LMβ1::Dendra2 band, although LMβ1 was detected (Fig. 3b). These data indicate the tagged protein to either be not secreted or the tag was proteolytically removed.

Next, the green signal from the edited clones were analysed using confocal microscopy. These images revealed the Dendra2 to be restricted to within the cytoplasm around the nucleus and translational organelles (Fig. 3c), and not in the characteristic LMβ1 distribution patterns beneath the cells. Processing with antibodies against LMα5, the major heterotrimeric partner of LMβ1 in A549 cells [13, 22], revealed a similar deposition pattern in the edited and control cells (Fig. 3d). Finally, cells were removed from coverslips using ammonium hydroxide to visualise only the ECM [19] (Fig. 3e). Although LMα5 was detected in the ECM, there was no detectable Dendra2 signal within the ECM of the 59B2 cells (Fig. 3e).

During routine culture of the edited cells, we became aware of a reduced growth rate in edited cells. Cell cycle analysis revealed a reduced proportion of the the LMβ1::Dendra2 cells in S phase and M Phase relative to A549 (8.6 ± 0.53% and 8.2 ± 0.52% reduction, respectively, both p < 0.01) (Fig. 3f).

### Discussion

These data demonstrate that adapting the Dendra2 tag from worms to mammalian cells led to Dendra2-tagged LMβ1 failing to be secreted or deposited, and causing detrimental effects to cell proliferation. There are many potential reasons for this lack of deposition, the most obvious being that addition of the 26 kDa tag inhibits either post-translational processing of the protein or interferes with LM trimerization. Indeed, the presence of LMα5 in the ECM in the edited cells suggests it is only the non-edited LMβ1 that is forming a heterotrimer with LMα5, and, as LM deposition is thought to be driven primarily by the α chain [1, 3], this seems most likely. Note that, based on the *C. elegans* and human fluorescent LMs [13, 15], we designed our construct to include a linker sequence in the LMβ1 C-terminus before the Dendra2, in an attempt to avoid this problem,. The ineffective secretion and deposition here suggests a fundamental difference in human LMβ1, but the reason for this difference is unclear.

Together, these data demonstrate that although tagging LMβ1 with Dendra2 at the C-terminus in human cells is possible, the protein is not deposited at detectable levels, which precludes its use for investigating BM assembly and dynamics. The additional complexity revealed here should be considered before any in vivo mammalian models are attempted.

## Limitations

Only a single LMβ1::Dendra2 clone was obtained, despite screening a large number of clones. This could be explained by the cell cycle defect in the edited cells. We predict the LAMB1 change has caused the cell division effects; however we cannot rule out an unknown off-target genome edit. To lessen this potential problem, we specifically chose to use the purified Cas9 protein which is known to reduce the frequency of off-target cleavage [23]. Irrespective of mechanism, the cell cycle effects would presumably be more severe in homozygous mutants, and which would present with a survival disadvantage, explaining the low number of clones obtained.

## Supplementary information

**Additional file 1.** gRNA sequences and potential off-target loci: Off-target sites as identified by Integrated DNA Technologies’ CRISPR-Cas9 gRNA Design Checker with mismatches (#MM) threshold set to 3. PAM = Protospacer adjacent motif sequence with only those recognised by purified Cas9 protein (NGG) shown. Off-target genes shown as—represent non-coding regions of the genome.

## Data Availability

All data generated or analysed during this study are included in this published article.

## Abbreviations

LM: Laminin
LCC domain: Laminin coiled-coil domain
CRISPR: Clustered regularly interspace short palindromic repeats
Cas9: CRISPR-associated protein 9
PBS: Phosphate buffered saline
3′UTR: 3′ Untranslated region
gRNA: Guide RNA
PCR: Polymerase chain reaction
ECM: Extracellular matrix
