# Supplementary material for: CRISPR-Cas9-mediated labelling of the C-terminus of human laminin β1 leads to secretion inhibition
Source: BMC Res Notes. 2020 Feb 21;13:90. doi: 10.1186/s13104-020-04956-z (PMC7035763; doi:10.1186/s13104-020-04956-z)
Supplement: Supplementary file 1 — Additional file 1. gRNA sequences and potential off-target loci: Off-target sites as identified by Integrated DNA Technologies’ CRISPR-Cas9 gRNA Design Checker with mismatches (#MM) threshold set to 3. PAM = Protospacer adjacent motif sequence with only those recognised by purified Cas9 protein (NGG) shown. Off-target genes shown as—represent non-coding regions of the genome. [file 13104_2020_4956_MOESM1_ESM.docx]

**gRNA sequences and potential off-target loci:** Off-target sites as identified by Integrated DNA Technologies’ CRISPR-Cas9 gRNA Design Checker with mismatches (#MM) threshold set to 3. PAM = Protospacer adjacent motif sequence with only those recognised by purified Cas9 protein (NGG) shown. Off-target genes shown as – represent non-coding regions of the genome.

| **gRNA** | **Sequence** | **PAM** | **#MM** | **Gene** | **Locus** |
| --- | --- | --- | --- | --- | --- |
| **1** | **ATAGCACATGCTTGTAACAG** | **AGG** | **-** | **LAMB1** | **chr7:-107923947** |
|  | ATAGCAC-TGTTTGTAACAG | AGG | 2 | - | chr20:-54489778 |
|  | CCAGCA-ATGCTTGTAACAG | GGG | 3 | - | chrX:-11679804 |
|  | ATGGAA-ATGCTTGTAACAG | AGG | 3 | - | chr2:-132853996 |
|  | GTAGC-CTTGCTTGTAACAG | AGG | 3 | - | chr3:-165621411 |
|  | AAAGCACATGCCTGAAACAG | AGG | 3 | PRAMEF18 | chr1:+13223043 |
|  | AAAGCACATGCCTGAAACAG | AGG | 3 | - | chr1:-13005892 |
|  | ATGGGACATGCTTGGAACAG | TGG | 3 | - | chr1:+201360309 |
|  | AAAGCACATGCCTGAAACAG | AGG | 3 | PRAMEF19 | chr1:+13368778 |
|  | ATAGCCCATCATTGTAACAG | GGG | 3 | - | chr1:-150169073 |
| **2** | **AAAAATGGCTGAGGTGAACA** | **AGG** | **-** | **LAMB1** | **chr7:-107923918** |
|  | AAAAATAGCAGAGGTGAACA | CGG | 2 | - | chr4:-185889429 |
|  | AAACATGGCTGA-GTGAACA | TGG | 2 | - | chrX:+72314675 |
|  | AATAATGGCTGAGGCTGAACA | CGG | 2 | - | chr22:+18396314 |
|  | AATAATGGCTGAGGCTGAACA | CGG | 2 | - | chr22:+18645927 |
|  | AATAATGGCTGAGGCTGAACA | CGG | 2 | - | chr22:-21349335 |
|  | AATAATGGCTGAGGCTGAACA | CGG | 2 | - | chr22:-18228229 |
|  | AATAATGGCTGAGGCTGAACA | CGG | 2 | - | chr22:-18723516 |
|  | GAACATGGCTGAGGTAAACA | TGG | 3 | - | chr6:+137830010 |
|  | AAAAATGCCTAGGGTGAACA | AGG | 3 | - | chr7:-61764339 |
|  | AATAAT-ACTGAGGTGAACA | TGG | 3 | - | chr15:+34472088 |
|  | AAAAATGCCTAGGGTGAACA | AGG | 3 | - | chr7:-62183366 |
|  | AATAATG-CTGCGGTGAACA | TGG | 3 | - | chr5:-9711216 |
|  | AATAATG-CTGAAGTGAACA | TGG | 3 | - | chr3:-192694445 |
|  | AATCATGGCTGAAGGTGAACA | AGG | 3 | - | chr11:+69188108 |
|  | ATACATGGCTG-GGTGAACA | AGG | 3 | - | chr2:-99577573 |
|  | AAACTTGGCTGA-GTGAACA | GGG | 3 | - | chr13:+20246396 |
|  | AATAAT-GCTGCGGTGAACA | TGG | 3 | - | chr2:+227929112 |
|  | AAAAATG-CTGCAGTGAACA | TGG | 3 | - | chr14:-90224265 |
|  | AAAAATG-CTGCAGTGAACA | TGG | 3 | - | chr8:-143542501 |
|  | AAAAATGCCTATGGTGAACA | AGG | 3 | - | chr5:-46283174 |
|  | AATAAT-GCCGAGGTGAACA | TGG | 3 | - | chr15:+76464689 |
|  | ATAAATG-CTGTGGTGAACA | TGG | 3 | - | chr4:-82865451 |
|  | GAAAATGGCAGAAGTGAACA | TGG | 3 | - | chr8:+60466821 |
|  | AATAAT-GCTGAGATGAACA | TGG | 3 | - | chr9:+2409871 |
|  | AAAAAT-GCCAAGGTGAACA | AGG | 3 | - | chr10:+128141546 |
|  | AATAATGG-TGAAGTGAACA | TGG | 3 | - | chr6:+39528402 |
|  | TAAAATGGC--AGGTGAACA | TGG | 3 | - | chr21:-13962038 |
|  | TAAAATGGC--AGGTGAACA | TGG | 3 | ANKRD20A9P | chr13:-18862737 |
|  | CAAAATGGCTCACGGTGAACA | AGG | 3 | - | chr5:+46013513 |
| **3** | **TTATATCCTTTAGGAGTGAA** | **CGG** | **-** | **LAMB1** | **chr7:+107923985** |
|  | AAATATCCTTTAGGAGTGAA | GGG | 2 | - | chr3:-188016088 |
|  | AAATATCCTTCAGGAGTGAA | GGG | 3 | - | chr14:-92066542 |
|  | AAATATCCTTCAGGAGTGAA | AGG | 3 | - | chrX:+72436480 |
|  | AAATATCCTTTAGGAATGAA | GGG | 3 | - | chr19:+33982373 |
|  | AAATATCCTTTAGGAATGAA | GGG | 3 | - | chr2:-46894629 |
|  | TAATATT-TTTAGGAGTGAA | AGG | 3 | - | chr2:-155009215 |
|  | TTCT-TCCTTTAGGAATGAA | AGG | 3 | - | chr18:-27661308 |
|  | CTATA-CTTTTAGGAGTGAA | GGG | 3 | - | chr21:-32994567 |
|  | TCATA-CCTTAAGGAGTGAA | TGG | 3 | - | chr13:+29911258 |
|  | CTAGATCCTTTAGGAGAGAA | AGG | 3 | - | chrX:+13414781 |
|  | TAAAATCCTTTAGGAGTTAA | GGG | 3 | - | chr9:+11381550 |
